# Pharmacokinetics and Toxicity Assessment of Artesunate and Its Metabolite, Dihydroartemisinin, in NOD Scid Gamma Mice

**DOI:** 10.3390/molecules31173111

**Published:** 2026-09-04

**Authors:** Prachi Agrawal, Sandeep K. Singh, Geoffrey Talmon, Sidharth Mahapatra, Daryl J. Murry

**Affiliations:** 1 Clinical Pharmacology Laboratory, Department of Pharmacy Practice and Science, University of Nebraska Medical Center, Omaha, NE 68198, USA; prachiagrawal0803@gmail.com (P.A.); sasingh@unmc.edu (S.K.S.); 2 Department of Pathology and Microbiology, University of Nebraska Medical Center, Omaha, NE 69198, USA; gtalmon@unmc.edu; 3 Fred and Pamela Buffett Cancer Center, University of Nebraska Medical Center, Omaha, NE 69198, USA; 4 Department of Biochemistry and Molecular Biology, University of Nebraska Medical Center, Omaha, NE 68198, USA; 5 Department of Pediatrics, Children’s Nebraska, University of Nebraska Medical Center, Omaha, NE 69198, USA

**Keywords:** pharmacokinetics, brain distribution, LC-MS/MS, maximum tolerated dose, toxicity

## Abstract

Artesunate (ART) is an FDA-approved antimalarial drug and is currently being repurposed for various solid tumor treatments. However, there are no safety or pharmacokinetic (PK) studies in mice exploring its potential application in murine brain tumor models. This study aims to delineate the tolerability and PK of ART in healthy NOD *scid* gamma (NSG) mice to inform future efficacy studies. The maximum tolerated dose (MTD) of ART following intraperitoneal (IP) administration was determined by a single-dose escalation method. PK and brain penetration of ART and its active metabolite, dihydroartemisinin (DHA), were analyzed using an optimized LC-MS/MS bioanalytical method, following single oral and IP doses (100 mg/kg). A 350 mg/kg IP dose was well tolerated with no evidence of systemic and organ-specific toxicities. Bioanalytical assay results were linear (R^2^ > 0.99) over a range of 5–1000 ng/mL. An optimized extraction method employing low sample volume improved analyte recovery from brain homogenate by 2-fold. PK studies showed rapid absorption with short elimination half-lives (t_1/2_: 8–22 min) and higher systemic and brain exposure for ART and DHA following IP administration over oral dosing. We conclude that ART is well-tolerated at high doses (350 mg/kg, IP) in NSG mice, although repeated doses may be necessary for therapeutic efficacy in brain tumors given its rapid elimination kinetics.

## 1. Introduction

ART is a semi-synthetic sesquiterpene lactone compound derived from the Chinese medicinal herb *Artemisia annua*, commonly referred to as sweet wormwood [1]. ART is approved by the World Health Organization and the FDA as first-line therapy for severe malaria [2,3], showing efficacy against susceptible and multidrug-resistant strains of Plasmodium falciparum [4]. Current FDA guidelines recommend an intravenous (IV) administration of 2.4 mg/kg of ART to adults at 0, 12, and 24 h. Subsequently, a once-daily IV infusion (6–120 s) of 2.4 mg/kg is administered for 5–7 days continuously or until the patient can tolerate oral antimalarial drugs [5]. In pediatric patients older than 6 months of age, a similar dosing regimen has been shown to produce comparable pharmacokinetic (PK) profiles and steady-state systemic exposures [5].

Beyond its anti-malarial efficacy, ART has demonstrated promise in treating various diseases, including cancer, diabetes, viral infections, and autoimmune disorders [1]. In cancer, ART has been shown to possess anti-neoplastic properties against lymphomas [6], high-grade gliomas [7], myelomas [8], and osteosarcomas [9]. Its cytotoxic action against cancer cells and tumors has been shown to be mediated by iron overload [7], depletion of glutathione stores [10], and induction of lipid ROS [7,10]. Additionally, ART inhibits the proliferation and invasion of tumor cells, induces cell death via apoptosis and ferroptosis [11,12,13], modulates the tumor microenvironment by enhancing the immune response [14], and inhibits tumor angiogenesis [15,16,17]. Furthermore, ART contributes to overcoming drug resistance in cancer cells through its multifaceted pharmacological actions. Notably, it inhibits lysosomal function of cancer cells [18], degrades TAZ (transcriptional coactivator with PDZ-binding motif) in EGFR-TKI resistant cells [19], inhibits proliferation in cisplatin-resistant bladder cancer cells [13], and inhibits JAK/STAT3 signaling pathways to reverse resistance in acute myeloid leukemia [20,21]. Other studies have elucidated the chemo-therapeutic activity of ART in glioblastoma tumors [22,23,24]. Clinically, artesunate has shown efficacy as an adjunct to chemotherapy in breast cancer (NCT00764036) [25], colorectal adenocarcinoma (ISCRTN05203252) [26], and recurrent glioblastomas (NCT02770378) [24,27].

Although these studies demonstrate favorable safety and efficacy profiles, a thorough understanding of PK and biodistribution is essential to repurpose ART for the treatment of brain tumors. In systemic circulation, ART is rapidly metabolized to its active isoforms, *α*- and *β*-DHA, by plasma and liver esterases. Between the two isoforms, *α*-DHA is more abundant than *β*-DHA. In humans, repeated administration of ART at a dose of 2.4 mg/kg produces a 5-fold higher AUC (area under the curve; exposure) for DHA. The elimination of ART (conversion to DHA) is significantly faster (180 L/h), resulting in a short half-life (0.3 h). On the other hand, DHA resides in the body longer (1.3 h), due to its slower clearance (32.3 L/h) by glucuronidation (UGT1A9 and UGT2B7) [4,5].

Despite being extensively studied in various species, including rats, dogs, monkeys, and humans, the existing toxicity, PK, and CNS penetration studies in mice are limited. Given the availability and common use of mice in pre-clinical studies, the aim of this study was to thoroughly characterize the MTD, systemic and organ-specific toxicity, and CNS penetration of ART and its active metabolite DHA in NSG mice to build a foundation for future studies in mouse models of CNS tumors.

## 2. Results

### 2.1. Bioanalysis

An ultra-high performance liquid chromatography tandem mass spectrometry (UHPLC-MS/MS) method was adopted from the literature and was successfully optimized to maximize the analyte and internal standard (IS) responses in positive atmospheric pressure chemical ionization (APCI) mode. The optimized MS parameters included an interface temperature of 200 °C; desolvation temperature of 355 °C; and heat block temperature of 200 °C with optimized nebulizing (3 L/min), heating (10 L/min), and drying (10 L/min) gas flow. The precursor ions were detected using a simple Q3 scan for both the analytes and IS. The most abundant precursor > product ion pairs detected using auto-optimization for ART, DHA, and IS was 221.15 *m*/*z* > 163.15 *m*/*z*, 221.15 *m*/*z* > 163.15 *m*/*z*, and 283.2 *m*/*z* > 247.1 *m*/*z*, respectively (Figure S1). The collision energy and voltage potential for the identification of precursor ions (Q1) and fragment ions (Q3) for ART, DHA, and IS were also optimized (Table S1). Despite having different molecular weight, the precursor ion *m*/*z* for ART and DHA was the same, due to the formation of a same mass ion (Figure S2), a phenomenon described by Naik and colleagues in 2005.

**Figure 1:**
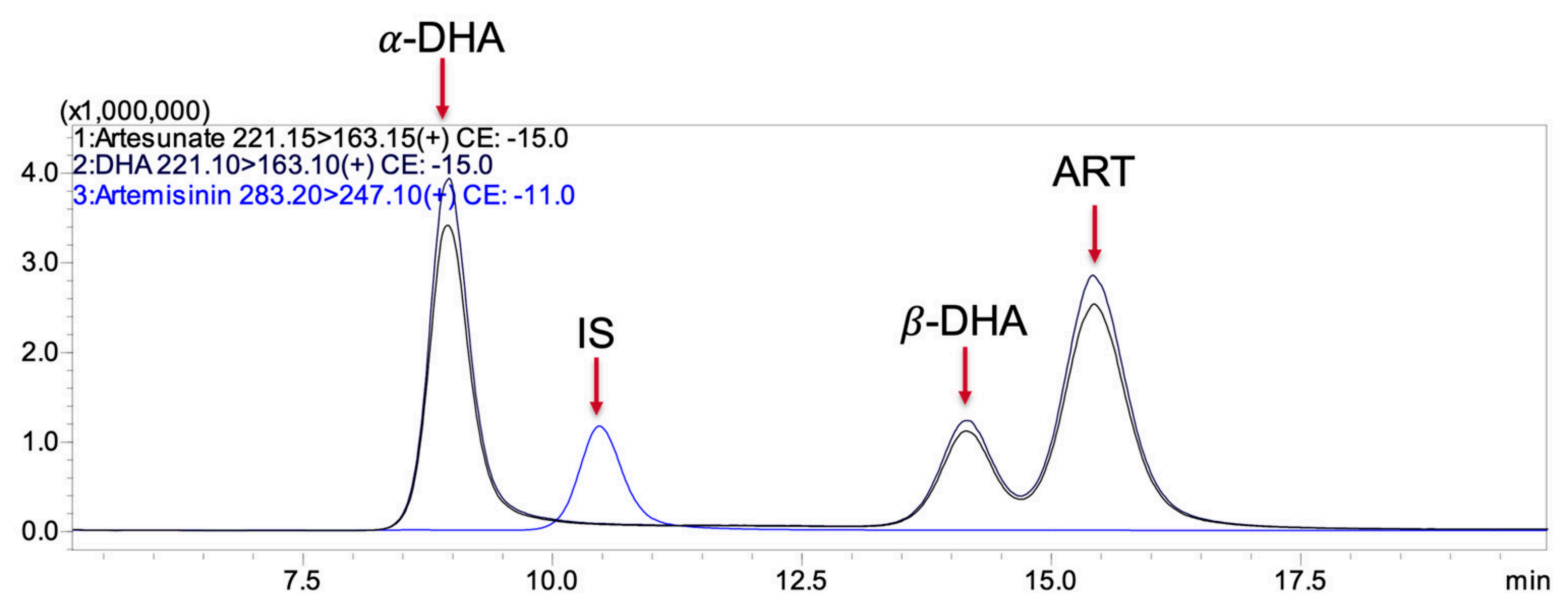
LC-MS/MS chromatogram of ART, *α*–DHA, *β*–DHA, and IS after spiking the analytes in mobile phase at a nominal concentration of 1000 ng/mL.

The mobile phase used for achieving the chromatographic separation was 0.1% acetic acid in water (aqueous phase, A: 44%) and 3:1 MeOH:ACN (methanol:acetonitrile, organic phase, B: 56%) supplemented to a Phenomenex Synergi Max-RP column at a constant flow rate of 0.2 mL/min, giving a column pressure of 1350–1400 psi. The mobile phase composition was isocratic throughout the analytical run (21 min), with an injection volume of 20 μL. Despite having the same *m*/*z*, the differences in physicochemical properties of ART, *α*-DHA, and *β*-DHA allowed separation on our bioanalytical column. The retention time for ART, *α*-DHA, *β*-DHA, and IS was 15.6 min, 9 min, 14.2 min, and 10.5 min, respectively (Figure 1). For estimating DHA concentrations across the studies, the peak area of *α*-DHA (DHA) was used, as *α*-DHA is ~3-fold more abundant than *β*-DHA. Using this method, the unknown concentrations of ART and DHA were accurately quantified. The assay was linear over the range of 5–1000 ng/mL with an R^2^ value of 0.992 and 0.995 for ART and DHA, respectively (Figure S3). Additionally, the assay was partially validated by establishing the accuracy and precision within each analytical run. The accuracy (%bias) and precision (%RSD) were with the acceptable range of ±15% of the nominal concentration for all quality control (QC) samples, except the lower limit of quantification (LLOQ), where the acceptance criterion was ±20% of the nominal concentration. The accuracy of ART and DHA was -1.5 to 14.5% and -3.4 to 6.3, and the precision was 1.0 to 5.0 and 1.4 to 4.4, respectively (Table S2).

The extraction method adopted from the literature was optimized to extract analytes from plasma and brain homogenate. The method demonstrated robustness in analyzing drug concentrations across diverse biological matrices with high extraction recovery (ER). The ER of compounds from mouse plasma when employing solid-phase extraction (SPE) was 72–83% (Table S3), well correlated with the literature (ART: 74–95% and DHA: 67–88%). However, SPE alone resulted in a poor extraction of compounds from brain homogenate (23–35%). Therefore, SPE was combined with protein precipitation to remove the interfering lipids/proteins, improving ER by 2-fold (55–71%). In contrast, the ER from mouse plasma remained relatively unchanged between the two methods (Table S3).

### 2.2. Maximum Tolerated Dose and Toxicity Assessment

Normal social and physical behavior prevailed for both male and female mice at ART doses of 60 and 108 mg/kg. At a dose of 195 mg/kg, social interaction among the animals diminished, their backs became slightly hunched, and their reflexes exhibited substantial reduction. However, the animals recovered their normal reflexes within 60 min post-dosing. Following IP administration at 350 mg/kg, transient physical responses including hunched back, isolation, and very slow reflexes even after external stimulation were observed immediately after dosing. However, these resolved spontaneously, and animals returned to their normal behavior within 60 min after dosing. Groups receiving no treatment or vehicle control exhibited normal behavior (Table S4).

No net weight gain or loss was observed in animals in the treatment group, suggesting that 350 mg/kg was well tolerated (Table 1). However, the sample size was not adequately powered to identify gender-based differences in toxicities.

Dose-limiting toxicities (DLTs) in clinical trials of ART have included anemia, leukopenia, neutropenia, weakness, and liver function abnormalities. Resultantly, we assessed for hematological and pathophysiological toxicities at the MTD in our treatment group. Complete blood count (CBC) analysis showed no difference between treatment groups in white blood cells, including lymphocytes and monocytes, red blood cells, or RBC indices (MCV or MCHC) compared to the reference range (sourced from VetScan HM-5 hematology system and Haney et al., 2018). The low lymphocyte counts observed in both control and treatment groups (10-fold lower than the lower limit of normal range) were expected given the immunocompromised nature of NSG mice. Although a significant difference in neutrophil counts was noted between groups, the values fell within the normal range. Similarly, although a statistically significant difference was observed between the two groups in mean corpuscular hemoglobin (MCH) levels, both groups had normal hemoglobin and mean corpuscular hemoglobin concentration (MCHC). A significantly higher platelet count was noted in the ART-treated mice, but this was not accompanied by any clotting events; notably these values were slightly higher than the upper limit for healthy mice (Table 2).

**Figure 2:**
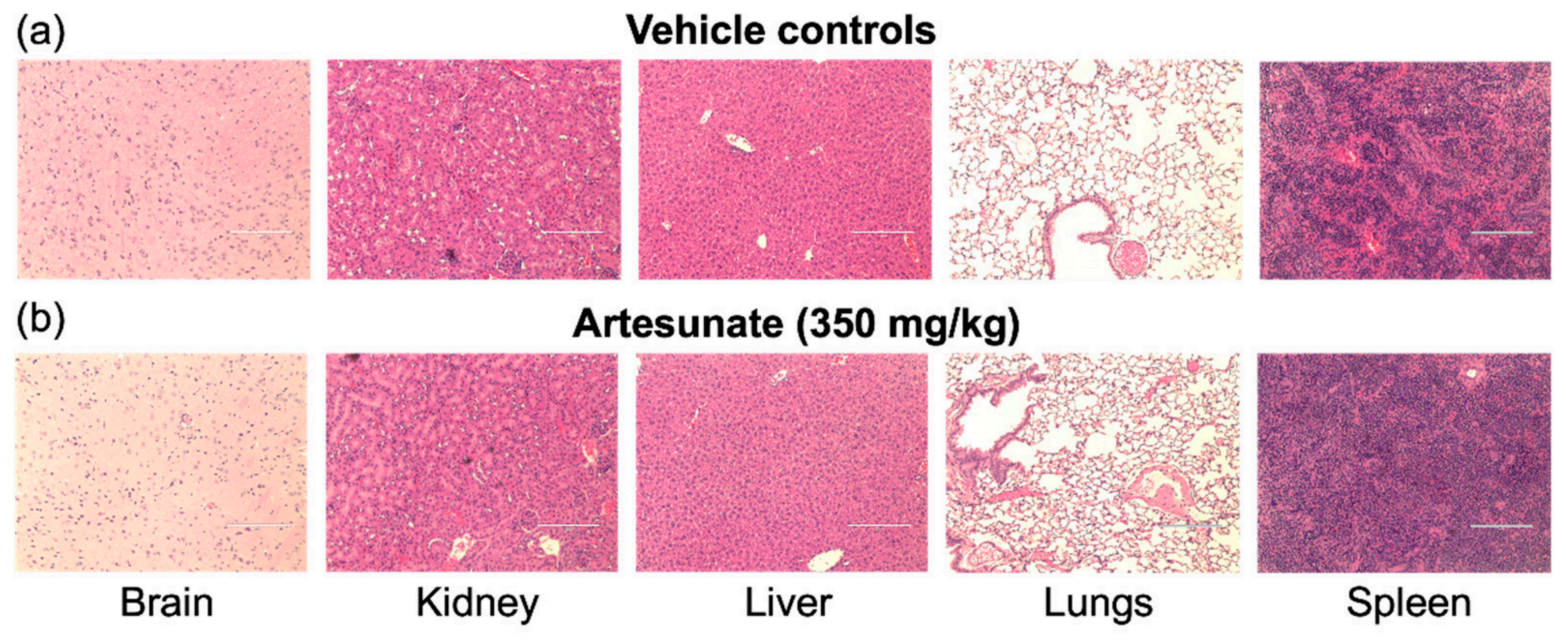
Histological evaluation (H&E staining) of brain, kidney, liver, lungs, and spleen from NSG mice administered with either (**a**) vehicle control or (**b**) ART dose of 350 mg/kg IP. Scale bar = 90 μm.

Of note, the CBC profile of animals receiving no treatment was similar to mice receiving vehicle control. Taken together, these findings did not reveal any hematologic toxicities at the MTD for ART in NSG mice. To complete our analysis for drug-induced tissue toxicity, tissue histology for the brain, kidney, liver, lungs, and spleen was compared between groups. No abnormalities were discerned from this comparative analysis (Figure 2). In summary, the overall toxicity evaluation indicated that the MTD of ART in healthy NSG mice was 350 mg/kg when administered intraperitoneally.

### 2.3. Pharmacokinetic Study

**Figure 3:**
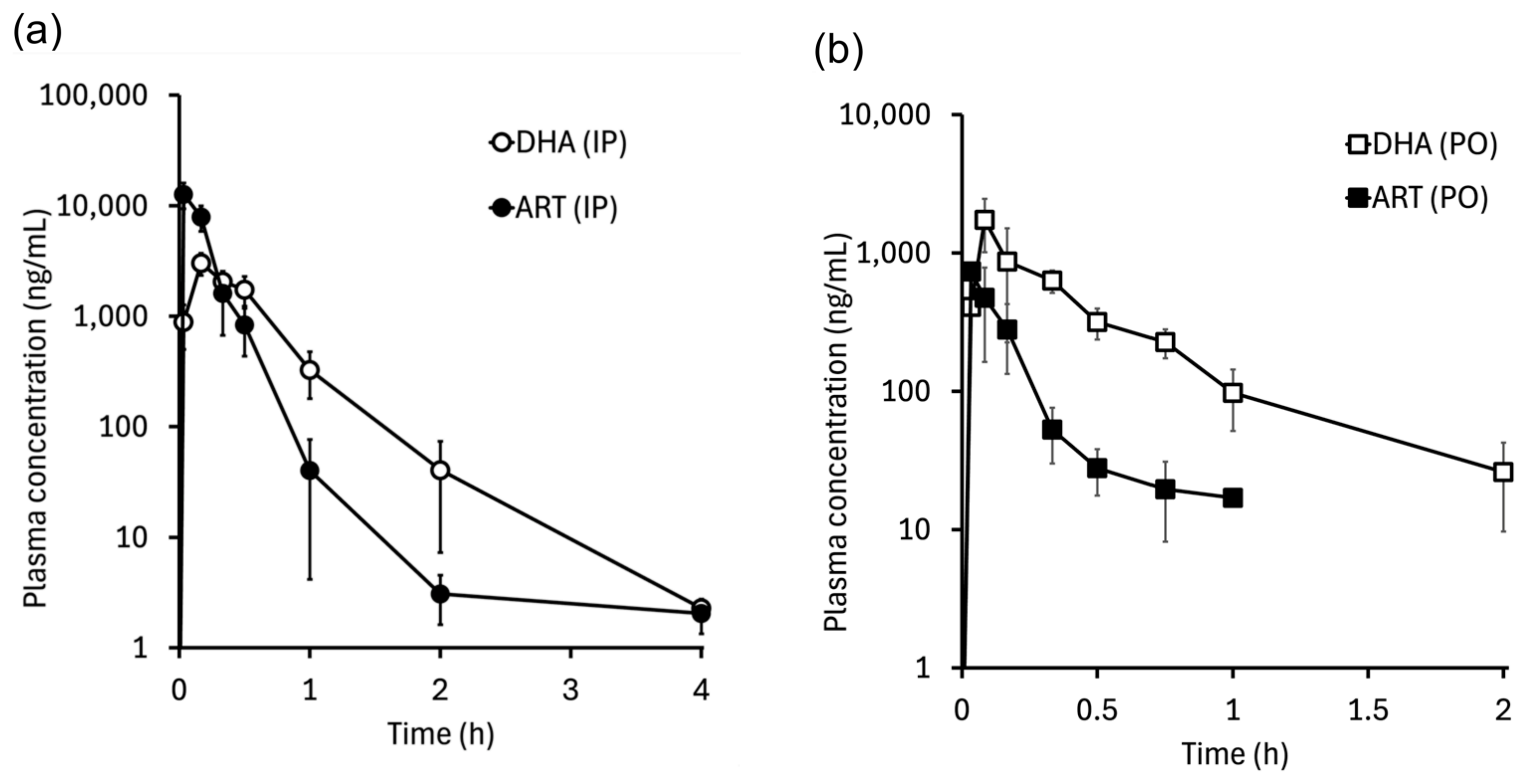
Plasma concentration-time profile of ART and DHA in healthy NSG mice after a single ART dose of 100 mg/kg given via (**a**) IP injection and (**b**) oral gavage (mean ± SD, *n* = 5).

Visual inspection of the plasma concentration time profile after IP dosing revealed that ART achieved maximum plasma concentration (C_max_) of 12827 ng/mL within 2 min (t_max_) post-administration. ART was rapidly converted to DHA by esterases to give a maximum DHA concentration of ~3000 ng/mL with a simultaneous reduction in ART concentration to ~8000 ng/mL, 10 min post IP dose (Figure 3a). The ART and DHA concentrations gradually decreased over time reaching 2 ng/mL and 2.3 ng/mL (below the limit of quantification, BLOQ) at 4 h post-dosing, respectively. The plasma concentrations were unquantifiable at 8 and 24 h.

ART is metabolized by plasma and tissue esterases. Therefore, systemic exposure of ART was also evaluated after a single PO dose to study its conversion to DHA by gut and liver esterases (Figure 3b). Systemic exposure of drug and metabolite after PO dosing was remarkably lower than after IP dosing, achieving maximum plasma concentration at 2 and 5 min post-dosing, respectively, with rapid elimination. In contrast, PO dosing produced a higher DHA C_max_ (1835 ng/mL) when compared to ART (726 ng/mL), with ART and DHA concentrations BLOQ at 2 and 4 h post-PO dosing, respectively.

The AUC_0-t_ for ART (3227.0 h×ng/mL) was 2-fold higher than for DHA (1635.8 h×ng/mL) after IP dosing. Subsequently, the apparent Vd_/F_ (17.4 L/kg) and CL_/F_ (32.8 L/h/kg) for ART were 2-fold lower than for DHA (33.2 L/kg and 61.8 L/h/kg, respectively). Despite the difference in clearance of the drug and its metabolite, both had elimination t_½_ values of 0.37 h (Table 3). On the other hand, a single PO dose exhibited a lower ART exposure (AUC_0−t_ 161.2 h×ng/mL) than DHA (AUC_0−t_ 578.7 h h×ng/mL), together with a 5.7-fold higher Vd_/F_ and ~3.6-fold higher CL_/F_.; this explained the shorter terminal t_½_ of ART (0.13 h) than DHA (0.28 h) (Table 3). The AUC_ratio_ (AUC_DHA_/AUC_ART_) values for IP and PO dosing were 0.5 and 3.6, respectively.

**Figure 4:**
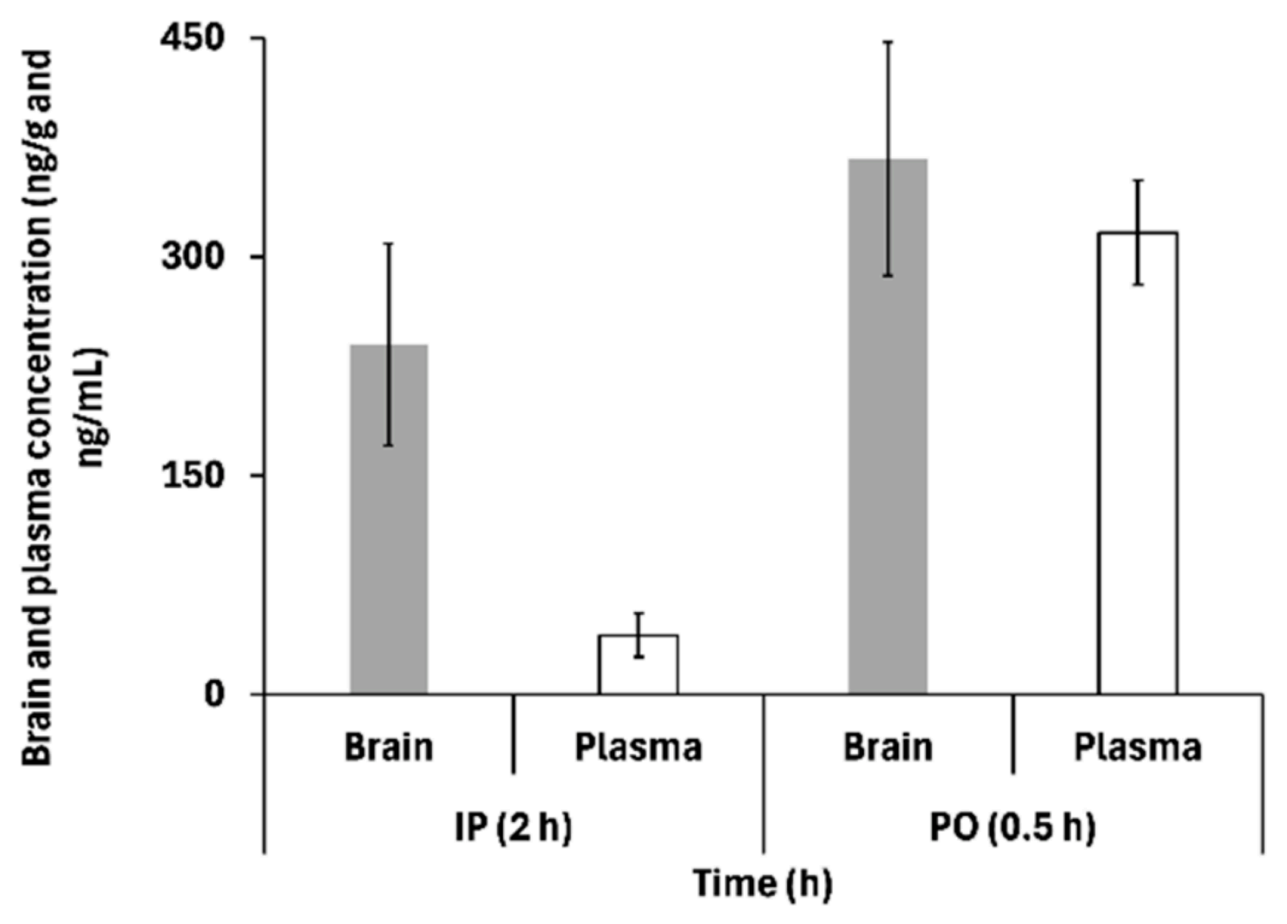
Brain and plasma concentration of DHA after a single 100 mg/kg IP and PO dose of ART in healthy NSG mice (mean ± SEM, *n* = 5).

A 100 mg/kg IP dose produced no quantifiable ART brain concentration at 2 h after dosing, versus a DHA brain concentration of 239.9 ± 154.6 ng/g. ART brain concentration after oral dosing was also BLOQ at 0.5 h, versus a DHA concentration of 366.8 ± 178.9 ng/g (Figure 4). At 8 and 24 h post-IP dose, as well as 1 and 4 h post-PO dose, the brain concentrations of ART and DHA were BLOQ.

## 3. Discussion

The primary objective of this study was to determine the MTD, pharmacokinetic profile, and tissue biodistribution of ART after PO and IP dosing. ART has been well-established for the treatment of malaria, but a renewed interest has risen based on burgeoning data demonstrating its anti-neoplastic properties in various cancers, including brain tumors. Thus, clinical trials have capitalized on these findings and demonstrated therapeutic efficacy for a strategy combining ART with current chemotherapeutic regimens [28,29].

Toxicity studies in humans indicate that ART has a broad therapeutic range. However, excessive dosage may lead to pancytopenia, leukopenia, weakness, melena, seizures, multiorgan failure, and even death [5,25,26]. In Sprague–Dawley rats, a MTD of 200–400 mg/kg was identified after a single IV injection of ART, with no deaths observed, at 240 mg/kg [30]. In Swiss albino mice, repeated ART doses of 300 mg/kg/day (oral) or 250 mg/kg/day (intramuscular) resulted in no neuronal cell death [31,32]. Since repeated dose toxicity after oral administration is already reported in the literature, the choice of route for maximum tolerated dose study was intraperitoneal. Our study determined the toxicity in NSG mice, revealing that a single IP dose of up to 350 mg/kg was well tolerated, as evidenced by the absence of hematological abnormalities and tissue histological changes (Table 2 and Figure 2) [33]. Furthermore, no weight loss or gain was observed in the animals (Table 1). Although 350 mg/kg impacted the behavior of animals, a rapid restoration of the physical behavior was observed (Table S4). Given that the bioavailability of drugs administered through the IP route is less than IV dosing (<100%) due to first-pass metabolism, the MTD following IP dosing is expected to be higher (350 mg/kg) than IV infusion. The MTD of ART after IV infusion was ≥240 mg/kg in mice, as reported by the FDA guidance of ART tolerability [34].

Renewed interest in repurposing ART has led several researchers to develop extraction techniques and bioanalytical assays for the quantification of the drug and its metabolites. However, the quantitation was limited to blood or plasma. An aim of this research was to extend the application of the published bioanalytical assay to a more complex biological matrix, i.e., the brain. The highest recovery and accuracy was obtained with slight modifications the methods developed by Naik and colleagues [35]. The optimized LC-MS/MS method was operated in positive APCI mode, with the highest separation and optimal peak shape observed with 0.1% acetic acid in water (44%) and MeOH: ACN (3:1, 56%), supplied at a flow rate of 0.2 mL/min. The response from the MS detector was linear over a concentration range of 5–1000 ng/mL at an injection volume of 20 μL. Adding a single-step protein precipitation prior to SPE enhanced the extraction recovery of the analytes from tissue homogenates by more than 2-fold. Lastly, the optimized bioanalytical assay and extraction method requires only 100 μL of the biological matrix, 5-fold less than that used in the literature [35].

The optimized LC-MS/MS and extraction method was applied to determine plasma and brain tissue concentrations in healthy NSG mice after a single IP dose of 100 mg/kg ART. When studying the PK behavior of ART, the PK of its major potent metabolite, DHA, was also studied [36]. IP and PO administration led to rapid absorption of the drug, giving a t_max_ of 2 min. Conversely, since DHA concentration was entirely dependent on the metabolism of ART, the maximum concentration of DHA was achieved 5 to 10 min after the ART dose. Additionally, the elimination of ART in humans (t_½_ 0.3 h) and mice (t_½_ 0.1–0.4 h) followed a similar trend. However, the elimination t_½_ of DHA is much more gradual in humans (1.3 h) than mice (t_½_ ~0.4 h). This variability can be explained by differences in the expression of metabolizing enzymes and transporters across species [37]. Rapid absorption and elimination of ART and DHA also support the findings of our MTD study, where animals displayed immediate physical responses upon dosing followed by restoration of normal behavior in less than 1 h at higher doses (195 and 350 mg/kg).

Gut and liver metabolism of the drug upon oral administration led to a higher systemic exposure of metabolite compared to the parent drug after PO dosing, producing an AUC_ratio_ (AUC_DHA_/AUC_ART_) of 3.6. In contrast, after IP administration, only plasma esterases were responsible for drug metabolism, producing an AUC_ratio_ of 0.5. Despite differences in AUC_ratio_, the AUC for the drug and active metabolite after a single IP dose were 20- and 2.8-fold higher than after a PO dose, respectively. Subsequently, higher systemic exposures suggest that IP route is preferable for the future efficacy studies, supporting the rationale for studying drug toxicity following IP administration.

Systemic concentrations are not the ideal marker for predicting therapeutic response in brain malignancies. Thus, brain concentrations were also quantified to understand its efficacy for repurposing in brain malignancies. ART concentrations were below the limit of quantification, while DHA levels in brain after 0.5 h post-PO dosing and 2 h post-IP dosing were 367 ng/g and 240 ng/g, respectively. The brain-to-plasma partition coefficient (K_p, brain_) of DHA was higher after IP dosing (K_p, brain_ 6 at 2 h) than PO dosing (K_p, brain_ 0.9 at 0.5 h), consistent with a higher systemic exposure after IP administration, suggesting a higher fraction of the drug and metabolite was available for biodistribution. These findings can be explained by its rapid plasma and gut metabolism and by the presence of efflux transporters, such as p-glycoprotein, on the intestinal wall and blood–brain barrier [38,39], thereby limiting its systemic bioavailability after PO dosing and biodistribution to the brain. Future studies will focus on studying brain concentrations immediately after dosing to better understand the brain exposure of the parent drug and metabolite in healthy mice and the brain tumor model.

Taken together, our PK studies suggest that once-daily dosing of ART in mice may not be sufficient to maintain therapeutic brain concentration throughout the day, especially if adjunctive treatment is being entertained for brain tumors. The MTD and PK studies support a strategy of increasing the administered dose and dosing frequency to achieve the required brain concentrations for therapeutic efficacy. Moreover, the FDA guidance for “Artesunate for injection” recommends thrice-daily dosing of ART for malaria. Based on our findings and the FDA guidance, a dose-ranging study would likely support more frequent dosing, especially in the first 24 h [5,34].

## 4. Materials and Methods

### 4.1. Chemicals and Materials

Artesunate (CAS number: 88495-63-0) and artemisinin (CAS number: 63968-64-9) were purchased from Thermo Scientific (Waltham, MA, USA). Dihydroartemisinin (CAS number: 71939-50-9) was obtained as a mixture of *α*-DHA and *β*-DHA from Tokyo Chemical Industry, Tokyo, Japan). LC-MS grade analytical solvents, ACN, MeOH, and acetic acid were obtained from Fisher Scientific (Fair Lawn, NJ, USA). Ultrapure water was prepared using a Barnstead GenPure water purification system (Thermo Scientific, Waltham, MA, USA). Mouse plasma was purchased from Innovative Research, Inc. (Novi, MI, USA).

### 4.2. Analytical Method

#### 4.2.1. Liquid Chromatographic and Mass Spectrometric (LC-MS/MS) Conditions

The bioanalysis was performed using UPLC-MS/MS system from Shimadzu Scientific Instruments (Columbia, MD, USA). Nexera UPLC was connected to a Shimadzu 8060 mass spectrometer, equipped with a dual ionizing source (DUIS, electrospray ionization; ESI and atmospheric pressure chemical ionization; APCI). The MS/MS system was operated at unit resolution in multiple reaction monitoring mode to maximize analyte sensitivity by eliminating background noise. The data analysis was performed using LabSolution software; Version 5.128 (Shimadzu Scientific Instruments, Columbia, MD, USA).

The assay parameters were adopted from literature with minor modifications [35]. The chromatographic separation of ART, DHA, and ARS was carried out on a Phenomenex Synergi Max-RP, 80 A, 75 mm × 2.0 mm, 4-micron (Torrance, CA, USA) column equipped with a Phenomenex guard column. Various organic (MeOH, ACN) and aqueous (0.1% acetic acid in water, 2–10 mM ammonium acetate buffer) mobile phase solvents were explored to achieve the separation of ART, ARS, and DHA based on the literature [35,40]. Each compound was analyzed individually on the LC-MS/MS, and retention time was noted and correlated with each analyte in the mixture. ARS, being a structural analog, was used as an IS to account for any analyte loss during sample preparation.

#### 4.2.2. Preparation of Stock, Calibration Standards, and Quality Control Samples

The primary stocks for analytes, ART and DHA, were prepared in methanol at a concentration of 1 mg/mL. Subsequently, these primary stocks were diluted to prepare a secondary stock at a concentration of 10 μg/mL (both ART and DHA). The secondary stock was serially diluted to prepare six working stocks (WS) for calibration standards, with concentrations ranging between 50–10,000 ng/mL, as previously described [41,42]. QC samples were analyzed within an analytical run to ensure consistent LC-MS response. The WS of QCs were prepared at concentrations of 50 ng/mL (LLOQ), 150 ng/mL (low quality control; LQC), 2000 ng/mL (medium quality control), and 7500 ng/mL (high quality control). In addition, a WS of IS (ARS) was also prepared in methanol at a concentration of 10 μg/mL. The WS for analytes (calibration standards and QCs) and IS was diluted 10-fold in the matrix of choice (mobile phase, plasma, or tissue homogenate) to give concentrations between 5 and 1000 ng/mL for calibration standard, 5, 15, 200, and 750 ng/mL for QCs, and 1000 ng/mL for IS [41]. The concentration of IS was kept constant throughout the studies to maintain consistency. The stocks were stored in −20 °C until their subsequent use. The bioanalytical assay was partially validated to ensure linearity, accuracy, and precision in each analytical run.

#### 4.2.3. Plasma and Tissue Sample Preparation

The extraction method employed a combination of protein precipitation and SPE. To a 100 μL aliquot of biological sample, 10 μL of the IS WS and 10 μL of 20 mg/mL sodium fluoride (NaF) solution were added. NaF was added to prevent ex vivo conversion of ART into DHA by inhibiting esterases. The sample underwent protein precipitation by adding 300 μL ice-cold ACN to release the analytes entrapped within the biological matrix. The precipitated samples were transferred to a 96-well Phree plate, where the precipitated phospholipids were trapped within the cartridge, allowing the organic supernatant along with the dissolved compounds to elute. To further purify the sample, SPE was performed using Oasis HLB cartridges (1 cc; 30 mg; Waters, Milford, MA, USA).

The SPE method was also adapted from the literature with minor modifications [35]. Briefly, the SPE method consisted of the following steps; priming, loading, washing, eluting, and reconstituting of the analytes. The sample preparation involved pre-acidification of the biological samples by diluting the 300 μL of eluent from protein precipitation with 3 mL of 1 M acetic acid, ensuring that the organic solvent concentration remained below 10%. The HLB cartridges were primed with 1 mL of methanol followed by 1 mL of 1 M acetic acid using a Cerex SPE processor (Varian Inc., Palo Alto, CA, USA). After conditioning, the acidified samples were loaded and passed through the cartridges under slight positive pressure. The cartridges were then washed with 1 mL of 1 M acetic acid followed by 1 mL of 20% methanol in 1 M acetic acid to remove the remaining salts or interfering moieties. Maximum recovery of analytes from the cartridge bed was ensured by passing 2 mL of highly lipophilic solvent (40% ethyl acetate in butyl chloride) through the cartridges and collecting it in clean glass vials. Finally, the eluent was evaporated under nitrogen, reconstituted with 100 μL of the mobile phase, vortexed, transferred to fresh LC vials, and analyzed using the validated LC-MS/MS method.

The extraction method for calibration and QC standards was the same as previously described, with a slight variation in the sample preparation method. In place of the study sample, a 100 μL aliquot of blank sample with a similar matrix was spiked with 10 μL of analyte WS to achieve various concentrations between 5 and 1000 ng/mL, while maintaining a consistent IS concentration of 1000 ng/mL.

ER was determined to measure the efficiency and reproducibility of the developed SPE procedure. ER was estimated for the highest calibration standard (nominal concentration 1000 ng/mL) by spiking ART to the pre-extracted blank biological matrix (post-extraction sample) and to blank biological matrix before extraction (pre-extraction). ER was calculated by comparing the peak area of analyte, metabolite, and IS in pre-extraction samples with the post-extraction samples (Equation (1)).

(1) $$Extraction\;recovery=\frac{Mean\;peak\;area\;of\;analyte\;spiked\;pre-extraction\;}{Mean\;peak\;area\;of\;analyte\;spiked\;post-extraction}\times 100$$

### 4.3. Maximum Tolerated Dose Study and Toxicity Evaluation

#### 4.3.1. Maximum Tolerated Dose Study

The MTD of ART was determined in both male and female NSG mice following IP administration. The animals were randomly divided into 3 groups of either five or three animals, namely the treatment group receiving ART (5 mice), the vehicle control group receiving blank vehicle (3 mice), and the control group receiving no treatment/vehicle (3 mice). The MTD was determined by a single dose escalation method where the animals received a starting safe dose of 60 mg/kg. The animals were observed for 48 h to ensure complete elimination of the drug before receiving the next higher dose. The dose was escalated 1.8-fold until toxicity was observed. Dose with no/reversible signs of toxicity was considered as the MTD. Animals received a single dose of 60, 108, 195, or 350 mg/kg ART. A 5% sodium bicarbonate (NaHCO_3_) solution in saline (0.9% sodium chloride) was used as a vehicle. Analyte solutions were heated to 50 °C to enhance the dissolution of ART, with fresh drug solution prepared on the day of the study.

#### 4.3.2. Toxicity Evaluation

Toxicity evaluation was performed by recording the weight of the animals, determining their social behavior, studying changes in their posture, and estimating the hematological and histological profile of the ART-treated group versus vehicle control groups.

The weights of the animals were recorded prior to dosing to serve as baseline. Subsequently, the animals were observed for the next 48 h post-treatment for any signs of toxicity, followed by a subsequent higher dose. A 48 h washout period allowed maximum elimination of the drug from mice, thereby minimizes the risk of toxicity due to drug accumulation. To account for any vehicle-related toxicities, the weights of both vehicle-treated and untreated animals were also recorded. The dose was considered toxic if more than 10% reduction in animal weight was observed. In addition to recording the weight of the animals, close observation was made of their social behavior (normal or isolated), body posture (normal or hunched), and movement (normal, reduced, or movement after stimulation).

Histological and hematological evaluation was performed to determine the toxicity of ART. For histological evaluation, vital organs (brain, lungs, liver, kidney, and spleen) were collected from the animals after receiving the MTD, washed with PBS, and fixed in 70% ethanol. The sectioning and H&E (hematoxylin and eosin) staining of vital organs were performed by the University of Nebraska Medical Center (UNMC) tissue sciences facility.

Hematological evaluation was performed to quantify various blood cells including RBC, WBC, platelets, lymphocytes, eosinophils, and basophils, as well as hemoglobin (Hb), between animals treated with drug versus vehicle control and/or untreated controls. CBC profiling was performed for all three treatment groups (ART-treated, vehicle-treated, and no doses). To generate the CBC profile, a small aliquot of fresh whole blood (40 μL) was collected in EDTA tubes within 2 h of dosing. The blood vial was kept on ice until analyzed. The samples were analyzed within 2 h after collection using the VetScan HM-5 Hematology system (Abaxis, Union City, CA, USA).

### 4.4. Preclinical Pharmacokinetics and Biodistribution Study

A preclinical PK and brain distribution study was conducted in healthy male and female NSG mice. The animals received a single IP or oral (PO) dose of 100 mg/kg ART. The vehicle used for dissolving the drug was the same as that employed in the MTD study (5% NaHCO_3_ in saline) and was freshly prepared on the day of the study. For each dosing group, animals were randomized into three groups of five mice for blood and brain collection at predetermined time points [42]. After IP dosing, blood aliquots from group 1 were collected at 2 min, 30 min, and 2 h (terminal); from group 2 at 10 min, 4 h, and 24 h (terminal); and from group 3 at 20 min, 1 h, and 8 h (terminal). For mice receiving PO doses, the blood from group 1 was collected at 2 min, 10 min, and 30 min (terminal); group 2 at 5 min, 20 min, and 1 h (terminal); and group 3 at 45 min, 2 h, and 4 h (terminal), post-dosing.

To minimize blood loss, only two 100 μL blood aliquots were collected from each animal, with the third aliquot collected at the terminal time point, when animals were euthanized and brains were harvested. Blood was collected in microcentrifuge tubes supplemented with 10 μL of NaF stock (20 mg/mL solution) and EDTA to prevent ex vivo conversion of ART into DHA. Immediately after collection, blood was spun down at 1000× *g* for 10 min to collect plasma. The brains were rinsed with phosphate buffered saline (PBS) to remove any blood clots and were homogenized. For homogenization, brain tissue was accurately weighed, diluted 5-fold with deionized (DI) water, and homogenized with a 5 mm stainless steel grinding ball using QIAGEN Tissue Lyser II (Louisville, KY, USA). The plasma and brain homogenates were stored at −20 °C until analyzed.

The plasma concentrations from IP and PO dosing groups were used to estimate the C_max_ and t_max_ of ART and DHA by visual inspection of the plasma concentration-time profile. Non-compartmental analysis using Phoenix WinNonlin (Version 8.5.2) was used to estimate the apparent volume of distribution (V_d_/F), AUC_0−t_, t_½_, and apparent clearance (CL/F).

## 5. Conclusions

In summary, this study optimized a bioanalytical assay and established the MTD and PK profiles of ART and its active metabolite DHA in healthy NSG mice, laying a critical pharmacological foundation for future efficacy studies. PK analysis revealed rapid absorption, distribution, and elimination of both analytes. Biodistribution studies revealed that ART, a known p-glycoprotein substrate, exhibited negligible brain penetration; however, DHA crossed the blood–brain-barrier in quantifiable concentrations before undergoing rapid elimination. To our knowledge, this is the first step towards the LC-MS/MS quantification of ART and DHA in brain tissues, enabled by the optimized brain tissue extraction method. MTD studies confirmed that doses up to 350 mg/kg were well-tolerated, establishing a favorable safety margin. Collectively, these findings support repeated high dosing to sustain therapeutic ART and DHA concentrations in brain over the dosing interval. Future work should leverage physiologically based pharmacokinetic modeling to guide dose and frequency optimization, with the goal of maximizing CNS penetration within the therapeutic window.

## Figures and Tables

**Table 1:** Normalized body weight of healthy NSG mice receiving either increasing doses of ART or equivalent volume of blank vehicle (vehicle control), or untreated controls.

Treatment	ART: 60 mg/kg	ART: 108 mg/kg	ART: 195 mg/kg	ART: 350 mg/kg
Artesunate (*n* = 5)	100.0 ± 0.0	100.9 ± 0.9	100.5 ± 2.9	100.0 ± 1.9
Vehicle control (*n* = 3)	100.0 ± 0.0	101.5 ± 1.4	102.9 ± 1.1	104.9 ± 1.4
Control (untreated, *n* = 5)	100.0 ± 0.0	101.3 ± 0.8	102.2 ± 2.1	102.0 ± 2.2

**Table 2:** Complete blood counts of healthy NSG mice treated with a single dose of 350 mg/kg ART and the mice receiving blank vehicle represented as mean (range), compared to the normal range.

Cell Type (Unit)	Vehicle Control (*n* = 3)	ART (350 mg/kg, *n* = 4)	Normal Range	*p*-Values
WBC (cells/μL)	0.9 (0.8–1)	1.2 (0.8–2.2)	0–15	0.46
LYM ^a^ (cells/μL)	0.3 (0.2–0.4)	0.3 (0.1–0.9)	3.4–7.4	0.99
MON (cells/μL)	0 (0–0.1)	0.1 (0–0.2)	0.0–0.6	0.14
NEU (cells/μL)	0.5 (0.5–0.6)	0.9 (0.8–1.1)	0.5–3.8	0.03
RBC (cells/L)	9.1 (8.4–9.6)	8.5 (7.8–9.1)	7.0–12.0	0.25
HBG (g/dL)	12.2 (11.3–13.4)	10 (6.8–11.7)	12.2–16.2	0.05
HCT%	42.1 (38.8–44.6)	38.8 (35.3–40.9)	35–45	0.20
MCV (fL)	46.3 (46.0–47.0)	45.5 (45–46)	45–55	0.15
MCH (pg)	13.5 (13.0–14.0)	11.8 (8.1–13.4)	11.1–12.7	0.01
MCHC (g/dL)	29 (27.8–30.1)	25.8 (17.6–29)	22.3–32	0.33
PLT (cells/L)	265.3 (234–314)	478 (433–535)	200–450	<0.01

^a^ Lymphocyte levels were low as immunocompromised strain was used for the study, WBC: White Blood Cell; LYM: Lymphocyte; MON: Monocyte; NEU: Neutrophil; RBC: Red Blood Cell; HBG: Hemoglobin; HCT: Hematocrit; MCV: Mean Corpuscular Volume; MCH: Mean Corpuscular Hemoglobin; MCHC: Mean Corpuscular Hemoglobin Concentration; PLT: Platelets.

**Table 3:** PK parameters of ART and DHA in healthy NSG mice after a single ART dose of 100 mg/kg administered intraperitoneally or orally (mean ± SD, *n* = 5) employing visual inspection (C_max_ and t_max_) and non-compartmental analysis.

Route of Administration	Parameters ^#^	ART	DHA
**Intraperitoneal dose (100 mg/kg)**	t_max_ (h)	0.033	0.167
	C_max_ (ng/mL)	12,826.6 ± 3112.0	2919.4 ± 807.3
	AUC_0-t_ (h×ng/mL)	3227.0 ± 907.6	1635.8 ± 193.6
	CL_/F_ (L/h/kg)	32.8 ± 8.1	61.8 ± 8.1
	Vd_/F_ (L/kg)	17.4 ± 5.2	33.2 ± 4.6
	t_½_ (h)	0.37 ± 0.02	0.37± 0.01
**Oral dose** **(100 mg/kg)**	t_max_ (h)	0.033	0.083
	C_max_ (ng/mL)	725.8 ± 132.9	1835.0 ± 686.3
	AUC_0-t_ (h×ng/mL)	161.2 ± 90.5	578.7 ± 150.6
	CL_/F_ (L/h/kg)	748.9 ± 358.5	174.5 ± 38.7
	Vd_/F_ (L/kg)	132.7 ± 65.2	71.0 ± 18.9
	t_½_ (h)	0.13 ± 0.04	0.28 ± 0.06

^#^ t_max_ time to achieve maximum concentration; C_max_ maximum concentration; AUC_0-t_ area under the curve from time 0 to t; CL_/F_ apparent clearance; Vd_/F_ apparent volume of distribution; t_½_ elimination half-life.

## Data Availability

Data are contained within the article and Supplementary Materials.

## Supplementary Materials

The following supporting information can be downloaded at: https://www.mdpi.com/article/10.3390/molecules31173111/s1, Figure S1: Mass spectra of (a) ART operated in positive APCI mode showing parent ion peak at 221.15 *m*/*z*, (b) DHA operated in positive APCI mode showing parent ion peak at 221.10 *m*/*z*, and (c) IS operated in positive APCI mode showing parent ion peak at 283.20 *m*/*z*. Figure S2: Chemical structures and molecular weight of ART, *α*- and *β*-DHA, and ARS along with the structure of precursor mass ion produced in the MS. Figure S3: Calibration curve of (a) ART and (b) DHA extracted from mouse plasma with concentrations ranging from 5 ng/mL to 1000 ng/mL and an R^2^ of 0.992 and 0.995, respectively. Table S1: Summary of molecular weight and optimized MS/MS parameters: precursor and fragment ion *m*/*z*, voltage potential, and collision energy for ART, DHA, and IS. Table S2: Accuracy (%bias) and precision (%RSD) for ART and DHA at 4 QC concentration levels (LLOQ, LQC, MQC, and HQC) in mouse plasma. Table S3: Extraction recoveries of ART, DHA, and IS from mobile phase, mouse plasma, and mouse brain homogenate employing SPE or a combination of SPE and protein precipitation at a nominal concentration of 1000 ng/mL. Table S4: Physical examination: Social behavior, body posture, and movement of animals receiving no treatment, vehicle control, and increasing intraperitoneal doses of ART (*n* = 3 or 5).
