# Supplementary material for: Pharmacokinetics and Toxicity Assessment of Artesunate and Its Metabolite, Dihydroartemisinin, in NOD Scid Gamma Mice
Source: Molecules. 2026 Sep 4;31(17):3111. doi: 10.3390/molecules31173111 (PMC13567107; doi:10.3390/molecules31173111)
Supplement: Supplementary file 1 [file molecules-31-03111-s001.zip › molecules-4490613-supplementary.pdf]

Supplementary information

Pharmacokinetics and Toxicity Assessment of Artesunate and Its Metabolite, Dihydroartemisinin, in NOD Scid Gamma Mice

Prachi Agrawal[1], Sandeep K. Singh[1], Geoffrey Talmon[2], Sidharth Mahapatra[3,4,5*], Daryl J. Murry[1,3,5*]

[1] Clinical Pharmacology Laboratory, Department of Pharmacy Practice and Science, University of Nebraska Medical Center, Omaha, NE 68198, USA; prachiagrawal0803@gmail.com (P.A.); sasingh@unmc.edu

[2] Department of Pathology and Microbiology, University of Nebraska Medical Center, Omaha, NE 69198, USA; gtalmon@unmc.edu

[3] Fred and Pamela Buffett Cancer Center, University of Nebraska Medical Center, Omaha, NE 69198, USA

[4] Department of Biochemistry and Molecular Biology, University of Nebraska Medical Center, Omaha, NE 68198, USA

[5] Department of Pediatrics, Children's Nebraska, University of Nebraska Medical Center, Omaha, NE 69198, USA

* Correspondence: sidharth.mahapatra@unmc.edu (S.M.); dj.murry@unmc.edu (D.J.M.);

Tel.: +1-(402)-955-8919 (S.M.); +1-(402)-559-3790 (D.J.M.)

[#] Corresponding authors:

Sidharth Mahapatra MD, Ph. D.<br>E-mail:<br>Pediatric Critical Care Medicine<br>Department of Pediatrics<br>University of Nebraska Medical Center<br>Omaha, NE 68198-7627<br>Phone: 402-955-8919	Daryl J Murry, PharmD<br>Email:<br>Pharmacy Practice and Science<br>Department of Pediatrics (courtesy)<br>University of Nebraska Medical Center<br>Omaha, Ne, 68198-6122<br>Phone: 402-559-3790

Figure S1

a)

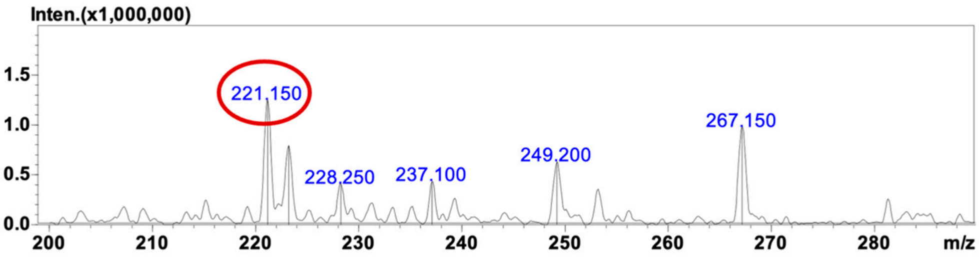

b)

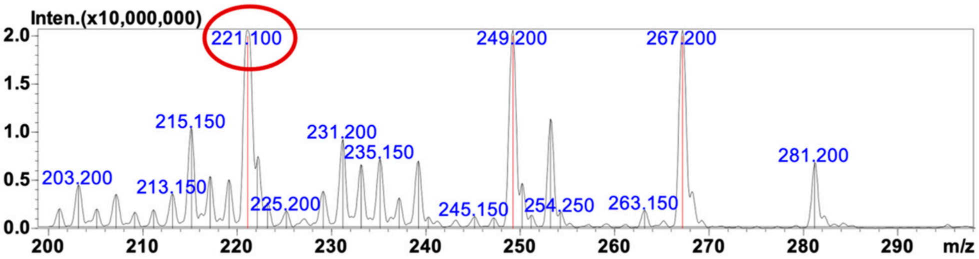

c)

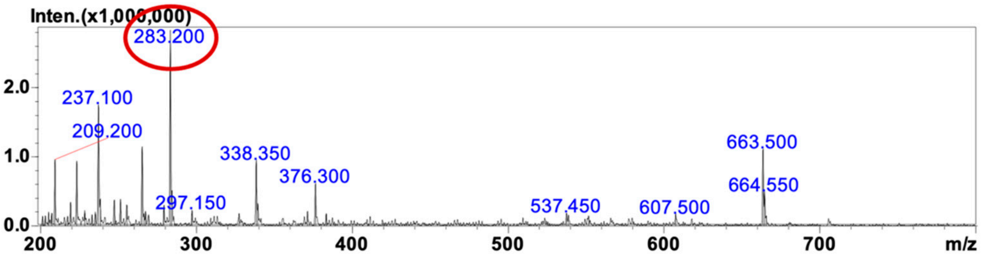

Figure S1. Mass spectra of (a) ART operated in positive APCI mode showing parent ion peak at 221.15 m/z, (b) DHA operated in positive APCI mode showing parent ion peak at 221.10 m/z, and (c) IS operated in positive APCI mode showing parent ion peak at 283.20 m/z.

Figure S2.

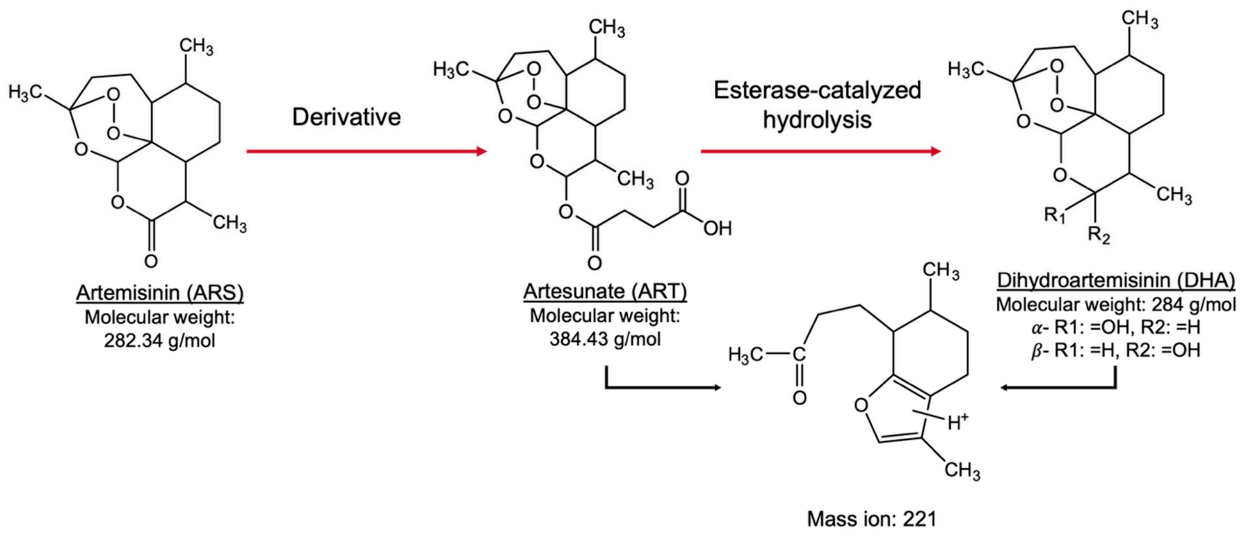

Figure S2. Chemical structures and molecular weight of ART, α- and β-DHA, and ARS along with the structure of precursor mass ion produced in the MS.

Figure S3.

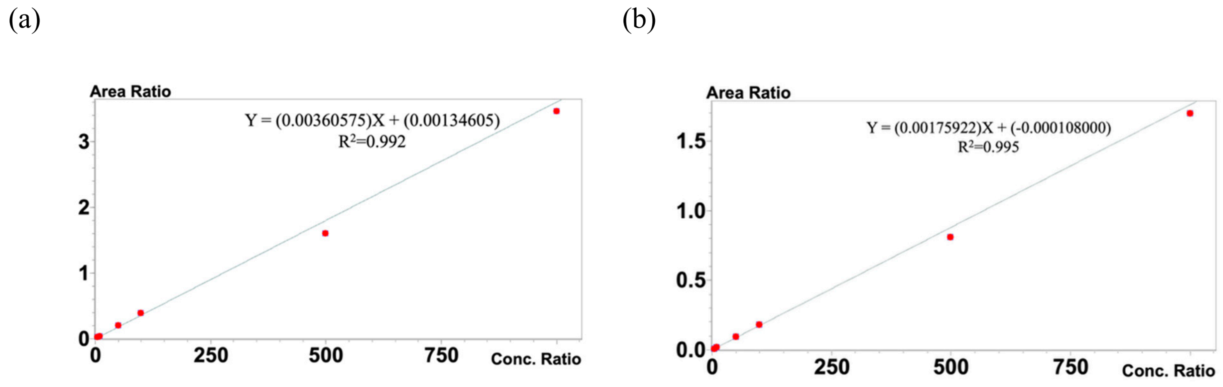

Figure S3. Calibration curve of (a) ART and (b) DHA extracted from mouse plasma with concentrations ranging from 5 ng/mL to 1000 ng/mL and an R^2 of 0.992 and 0.995, respectively

Table S1. Summary of molecular weight and optimized MS/MS parameters: precursor and fragment ion m/z, voltage potential, and collision energy for ART, DHA, and IS.

Compound	Molecular weight	Precursor (Q1) > fragment (Q3) ion m/z	Q1 (V)	Collision energy (V)	Q3 (V)
ART	384.43 g/mol	221.1 > 163.1	-13	-15	-29
DHA	284 g/mol	221.1 > 163.1	-20	-15	-20
IS	282.34 g/mol	283.2 > 247.1	-13	-11	-26

Table S2: Accuracy (%bias) and precision (%RSD) for ART and DHA at 4 QC concentration levels (LLOQ, LQC, MQC, and HQC) in mouse plasma

QC	ART		DHA	
	% Bias	% RSD	% Bias	% RSD
LLOQ	-1.5	1.0	-3.4	1.4
LQC	1.5	1.1	5.6	1.8
MQC	14.5	1.3	6.3	2.6
HQC	10.5	5.0	1.5	4.4

Table S3. Extraction recoveries of ART, DHA, and IS from mobile phase, mouse plasma, and mouse brain homogenate employing SPE or a combination of SPE and protein precipitation at a nominal concentration of 1000 ng/mL.

Matrix type	Extraction method	ART (%)	DHA (%)	IS (%)
Mobile phase	N/A	100	100	100
Plasma	SPE	81	83	72
	Protein precipitation + SPE	82	79	76
Brain	SPE	23	35	23
	Protein precipitation + SPE	55	71	55

Table S4. Physical examination: Social behavior, body posture, and movement of animals receiving no treatment, vehicle control, and increasing intraperitoneal doses of ART (n = 3 or 5)

Physical examination	Control (untreated, *n*=3)	Vehicle control, IP (*n*=3)	ART: 60 mg/kg, IP (*n*=5)	ART: 108 mg/kg, IP (*n*=5)	ART: 195 mg/kg, IP (*n*=5)	ART: 350 mg/kg, IP (*n*=5)
Social behavior	Normal	Normal	Normal	Normal	Somewhat isolated	Isolated
Body posture	Normal	Normal	Normal	Normal	Slightly hunched	Hunched
Movement	Normal	Normal	Normal	Normal	Slow reflex	Slow reflex even after stimulation
